# PTBP1 modulates osteosarcoma chemoresistance to cisplatin by regulating the expression of the copper transporter SLC31A1

**DOI:** 10.1111/jcmm.15183

**Published:** 2020-03-24

**Authors:** Cheng Cheng, Qiuyue Ding, Zhicai Zhang, Shangyu Wang, Binlong Zhong, Xin Huang, Zengwu Shao

**Affiliations:** ^1^ Department of Orthopedics Union Hospital Tongji Medical College Huazhong University of Science and Technology Wuhan China

**Keywords:** chemoresistance, osteosarcoma, PTBP1, SLC31A1

## Abstract

Chemoresistance is the main obstacle of treatment in patients with osteosarcoma. RNA‐binding protein PTBP1 has been identified as an oncogene in various cancers. However, the role of PTBP1 in osteosarcoma, especially in chemoresistant osteosarcoma, and the underlying mechanism remain unclear. In this study, we aimed to explore the functions of PTBP1 in chemoresistance of osteosarcoma. We found that PTBP1 was significantly increased in chemotherapeutically insensitive osteosarcoma tissues and cisplatin‐resistant osteosarcoma cell lines (MG‐63_CISR_ and U‐2OS_CISR_) as compared to chemotherapy‐sensitive osteosarcoma tissues and cell lines. Knock‐down of PTBP1 can enhance the anti‐proliferation and apoptosis‐induced effects of cisplatin in MG‐63_CISR_ and U‐2OS_CISR_ cells. Moreover, PTBP1 knock‐down significantly up‐regulated the expression of the copper transporter SLC31A1, as indicated by transcriptome sequencing. Through RNA immunoprecipitation, dual‐luciferase reporter assay and RNA stability detection, we confirmed that PTBP1 binds to SLC31A1 mRNA and regulates the expression level of SLC31A1 by affecting mRNA stability. Additionally, SLC31A1 silencing abrogated the chemosensitizing effect of PTBP1 knock‐down in MG‐63_CISR_ and U‐2OS_CISR_ cells. Using a nude mouse xenograft model, we further confirmed that PTBP1 knock‐down enhanced chemoresistant osteosarcoma responsiveness to cisplatin treatment in vivo. Collectively, the present study suggests that PTBP1 is a crucial determinant of chemoresistance in osteosarcoma.

## 1 INTRODUCTION

Osteosarcoma is the most common primary malignant bone tumour in children and adolescents.^1^ For osteosarcoma treatment, the application of neoadjuvant chemotherapy drugs, including cisplatin, doxorubicin and methotrexate, has improved the limb salvage rate and raised the long‐term survival rate of patients to approximately 70%.^2^, ^3^ Cisplatin exerts anti‐cancer effects by crosslinking with the purine bases on DNA and interfering with DNA repair mechanisms, subsequently causing DNA damage and eventually inducing apoptosis of cancer cells.^4^, ^5^ Although this treatment strategy is effective, the development of cisplatin resistance has emerged as a major cause of chemotherapy failure, resulting in overall survival stagnation over the past three decades.^6^, ^7^, ^8^ Therefore, overcoming the chemoresistance of osteosarcoma and exploring potential molecular mechanisms have become key issues for improving the survival rate of osteosarcoma patients.

Cisplatin resistance attributes to a variety of factors, including abnormal genetic alterations, tumour microenvironment changes, epigenetic abnormalities and cancer stem cells.^9^, ^10^, ^11^, ^12^ It is noteworthy that drug accumulation disorder has emerged as a crucial contributor in the cisplatin resistance.^13^ Traditionally, platinum drugs penetrate cell membranes mainly through passive diffusion, but recent studies have found that ATP‐consuming active transport weighs more in platinum drug transport, and copper transporters (CTRs) are responsible for this transport pattern.^14^, ^15^ Copper transporters are mainly distributed in cell membranes, endoplasmic reticulum, lysosomes and other organelles, and the main function of CTRs is to transport copper and maintain intracellular copper homeostasis.^16^ CTRs involved in platinum drug transport mainly depend on copper transporter 1 (SLC31A1), copper transporter 2 (SLC31A2), ATPase copper transporting alpha (ATP7A) and ATPase copper transporting beta (ATP7B). Among them, SLC31A1 is the major member that affects the intracellular concentration of platinum drugs and mediates the uptake of 60%~70% cisplatin and 30%~40% carboplatin in cells.^17^ Previous studies have demonstrated that the expression level of SLC31A1 is associated with the chemoresistance of cancers. Yong et al found that oleandrin increases the chemosensitivity of osteosarcoma by inhibiting the degradation of copper transporter 1.^18^ Lv et al^19^ corroborated that down‐regulation of copper transporter 1 is responsible for the cisplatin resistance in ovarian cancer. Takeda et al^20^ revealed the relationship between the expression level of copper transporter 1 and chemoresistance in triple‐negative breast cancer. However, the role of SLC31A1 in cisplatin‐resistant osteosarcoma remains unclear.

Post‐transcriptional regulation, as a key regulation mode that affects various biological behaviours of tumours, has been extensively studied by researchers.^21^, ^22^ Post‐transcriptional regulation depends on the RNA‐binding proteins (RBPs), which directly bind to target RNA and affect the transport, stability, splicing and degradation of RNA, thereby regulating the expression of target genes and playing a role in pro‐cancer or anti‐cancer process.^23^, ^24^ PTBP1 is a newly discovered RNA‐binding protein which specifically binds to polypyrimidine sequences on RNA and regulates the expression of downstream target genes with different or even opposing functions.^25^ Increasing evidence has demonstrated that PTBP1 plays a critical role in the malignant characteristics of tumours and may be a target for cancer treatment. Xie et al^26^ found that PTBP1 promotes the proliferation and metastasis of bladder cancer cells by selective splicing of MEIS2 and PKM. Li et al^27^ demonstrated that PTBP1 promotes the migration and invasion of lung cancer cells by enhancing the skipping of exon11a of the MENA precursor gene. Jo et al^28^ revealed that PTBP1 promotes metastasis of colorectal cancer by binding ATG10 mRNA and suppressing its expression. More interestingly, PTBP1 is associated with the response of cancer cells to chemotherapeutic drugs. Cheng et al^29^ found that PTBP1 ablation could overcome resistance of colon cancer cell to vincristine and oxaliplatin through glycolytic regulation. Cui et al^30^ confirmed that PTBP1 affects the apoptosis of cancer cells induced by chemotherapeutic drugs by regulating the expression of MCL. Besides, former studies have uncovered that PTBP1 undergo regulation of non‐coding RNAs in the progression of various cancers.^31^, ^32^ Currently, the status of PTBP1 in osteosarcoma and the underlying regulatory mechanisms of PTBP1 affect biological characteristics, especially the chemotherapeutic response of osteosarcoma cells, and have not been clearly reported.

In the present study, we aim to investigate the role of PTBP1 in cisplatin‐resistant osteosarcoma; moreover, we sought to address the detail mechanisms on how PTBP1 affects the cisplatin resistance of osteosarcoma. We first confirmed that PTBP1 expression was increased in human osteosarcoma tissues, especially tissues with a poor response to chemotherapy. Then, we demonstrated that PTBP1 knock‐down enhanced the chemosensitivity of cisplatin‐resistant osteosarcoma cells to cisplatin by directly up‐regulating the expression level of SLC31A1. Furthermore, we established a cisplatin‐resistant osteosarcoma xenograft model in nude mice and exhibited that PTPB1 silencing could significantly enhance the inhibitory effect of cisplatin on cisplatin‐resistant osteosarcoma. Taken together, our results suggest that PTPB1 is a novel target for overcoming the cisplatin resistance of osteosarcoma.

## 2 MATERIALS AND METHODS

### 2.1 Patients and clinical samples

Twenty‐five osteosarcoma patients who underwent tumour resection in the department of orthopaedics of Wuhan Union Hospital were selected. All patients received a cisplatin‐based chemotherapy regimen before surgery. Tumour tissues and corresponding adjacent normal tissues were frozen in liquid nitrogen within 30 minutes after isolation. The collected specimens were obtained with the informed consent of the patients or the patient's parents. The chemotherapeutic response of cancer tissue was assessed by an expert panel of pathologists. The good chemotherapy response (GR) was defined as the necrosis rate higher than 90% and the poor response (PR) as the necrosis rate <90%. This study was approved by the Ethics Committee of Tongji Medical College and complied with the Declaration of Helsinki.

### 2.2 Cell culture, transfection and treatment

Human osteosarcoma cell lines MG‐63, U‐2OS and human osteoblast cell line NHOst were obtained from ScienCell Research Laboratories and cultured in DMEM/F12 medium (Gibco) containing 10% foetal bovine serum (Gibco) and 1% penicillin‐streptomycin (Biosharp) at 37°C and 5% CO_2_.

PTBP1 short hairpin RNA (sh‐PTBP1) and control vector plasmids (sh‐control) were purchased from Genechem to specifically knock‐down PTBP1. The sh‐PTBP1 #1 sequence was 5′‐AGCCCATCTACATCCAGTT‐3′; the sh‐PTBP1 #2 sequence was 5′‐CAACGTCAAGTACAACAAT‐3′; the sh‐PTBP1 #3 sequence was 5′‐TCGTCAAAGGATTCAAGTT‐3′, and the sh‐control sequence was 5′‐ TTCTCCGAACGTGTCACGT‐3′. Small interfering RNA specific for SLC31A1 (si‐SLC31A1) and negative control (si‐NC) were purchased from Ribobio. The si‐SLC31A1 sequence was 5′‐CUGCGUAAGUCACAAGUCA dTdT‐3′. Lipofectamine 3000 (Invitrogen) was used to conduct cell transfections in accordance with the manufacturer's guidelines.

For cisplatin‐resistant cell lines generation, we prepared 50 mmol/L cisplatin solution and diluted it to 1~50 μmol/L for use. In vitro experiments, we used completed medium containing 0.01% DMF as control group and 1 μmol/L cisplatin treated cells for 48 hours as cisplatin treatment group.

### 2.3 Bioinformatics analysis

Data sets of gene expression profiles and clinical follow‐up data of six common tumours, bladder cancer (BLCA), colon cancer (COAD), renal clear cell carcinoma (KIRC), hepatocellular carcinoma (LIHC), lung adenocarcinoma (LUAD) and gastric cancer (STAD), were downloaded from the TCGA database (http://cancergenome.nih.gov/). Data were converted, managed and analysed by the edgeR package in R (version 3.4.1) to compare the expression of PTBP1 in the above tumours and normal tissues. The KM‐plot website (http://www.kmplot.com/) was used to identify the correlation between PTBP1 level and prognosis in the TCGA data sets.

### 2.4 Generation of cisplatin‐resistant cell lines

According to a previous study,^4^ cisplatin‐resistant (CISR) sub‐lines MG‐63_CISR_ and U‐2OS_CISR_ were derived from MG‐63 and U‐2OS cells by continuous stimulation with increasing concentrations of cisplatin. Initially, 1 μmol/L cisplatin was used to treat MG‐63 and U‐2OS cells for 72 hours, and then, the medium was replaced with cisplatin‐free medium to restore the cells to their previous state. Thereafter, the low to high dosage of cisplatin (1~50 μmol/L) was used to stimulate cells. CISR cells were obtained when 30 μmol/L cisplatin killed cells <25%.

### 2.5 Cell viability assay

Cell viability was determined by Cell Counting Kit‐8 (CCK‐8, Dojindo) assay and colony formation assay. For the CCK‐8 assay, cells with different treatments were cultured in 96‐well plates at 5000 cells per well. After the indicated times, exchanged for serum‐free medium and added 10 μL CCK‐8 solution into each well. The absorbance at 450 nm was measured by a microplate reader (Thermo Scientific) after incubation at 37℃ for 2 hours.

The transfected and control group cells were seeded in 6‐well plates at 500 cells per well, treated with 1 μmol/L cisplatin for 48 hours upon cell adherence and then cultured under standard conditions, and the culture medium was changed every 3 days. Fourteen days later, the cells were fixed with polyformaldehyde and stained with 0.1% crystal violet (Beyotime). The number of colonies was counted with ImageJ software.

### 2.6 Flow cytometry

An Annexin V‐FITC apoptosis kit (BD Biosciences) was used to determine the number of apoptotic cells according to the manufacturer's instructions. Briefly, cells were harvested and washed twice with cold PBS and resuspended in 500 μL of binding buffer. The cell samples were incubated with Annexin V for 10 minutes in the dark and then incubated with FITC for 5 minutes. Flow cytometric analysis was performed with a FACS Calibur flow cytometer (BD Biosciences) and WINMDI 2.8 software.

### 2.7 Quantitative real‐time PCR

Total RNA was extracted with TRIzol reagent (Beyotime) and used to synthesize cDNA with a PrimeScript™ II First‐Strand cDNA Synthesis Kit (Takara). qRT‐PCR primers were generated by Sangon Biotech. qRT‐PCR was carried out using a SYBR Primer‐Script RT‐PCR kit (Takara) with a CFX96 Touch quantitative PCR system (Bio‐Rad). To obtain more reliable results, we selected β‐actin and GAPDH as the internal reference genes through geNorm software evaluation. We calculated the geometric mean of the Ct value of two internal reference genes and then analysed relative gene expression by the 2^−ΔΔCt^ method. Primers for PTBP1 were 5′‐GCTGCACCTCTCCAACATCC‐3′ (forward) and 5′‐GTCGTGGTTGTGCAGGTCAA‐3′ (reverse); primers for SLC31A1 were 5′‐GGGGATGAGCTATATGGACTCC‐3′ (forward) and 5′‐TCACCAAACCGGAAAACAGTAG‐3′ (reverse); primers for β‐actin were 5′‐AGAGCTACGAGCTGCCTGAC‐3′ (forward) and 5′‐AGCACTGTGTTGGCGTACAG‐3′ (reverse); primers for GAPDH were 5′‐GAAGGTGAAGGTCGGAGTC‐3′ (forward) and 5′‐GAAGATGGTGATGGGATTTC‐3′ (reverse).

### 2.8 Western blot analysis

Total proteins were extracted from cells using lysis buffer supplemented with the complete protease inhibitor cocktail (Abcam). The following primary antibodies were used for Western blot analysis: anti‐PTBP1 (ab133734, Abcam), anti‐SLC31A1 (ab129067, Abcam), anti‐Cleaved Parp antibody (ab32064, Abcam), anti‐Cleaved Caspase‐3 antibody (ab2302, Abcam), anti‐Bax antibody (ab32503, Abcam), anti‐Bcl‐2 antibody (ab32124, Abcam) and anti‐β‐actin antibody (ab8226, Abcam). The detailed steps of the assay have been described in our previous paper.^33^ Briefly, the protein concentration was determined by BCA method, and then, total protein lysates were fractionated using SDS‐PAGE and transferred onto PVDF membranes. Membranes were blocked with 5% non‐fat milk and incubated with the above primary antibodies. Horseradish peroxidase‐conjugated secondary antibody was used to develop blots. The grey value was measured by ImageJ software.

### 2.9 Transcriptome sequencing and gene enrichment analysis

About 3 μg of high‐quality total RNA from MG‐63_CISR_ or U‐2OS_CISR_ cells transfected with sh‐PTBP1 or sh‐control was used as the sample preparations and sent to a commercial company for transcriptome sequencing (Genergy Inc). The DEGseq package was used to identify differentially expressed genes between the control group and the sh‐PTBP1 group with parameters of |log_2_FoldChange| ≥ 1 and *P* value < .05. Gene Ontology analysis and KEGG Pathway analysis of differentially expressed genes were conducted with the clusterprofiler package in R.

### 2.10 RNA immunoprecipitation

RNA immunoprecipitation was performed using the Magna RIP RNA‐Binding Protein Immunoprecipitation kit (Millipore) according to the manufacturer's instructions. Briefly, cells were washed with cold PBS and lysed in RIP lysis buffer. A/G immunomagnetic beads and anti‐PTBP1 antibody or anti‐IgG (ab172730, Abcam) were premixed in immunoprecipitation buffer to immunoprecipitate endogenous PTBP1‐RNA complexes. After immunoprecipitated complexes were washed, they were treated with proteinase K. RNA isolation was conducted by the phenol‐chloroform method. The purified RNA was used for qPCR to measure the level of SLC31A1 mRNA binding with PTBP1 protein. Results are presented relative to IgG immunoprecipitation.

### 2.11 RNA stability

To evaluate the effect of PTBP1 on the stability of SLC31A1 mRNA, we detected the half‐life of SLC31A1 mRNA. About 5 μg/mL actinomycin D was used to treat cells, which were subsequently collected at 0, 2, 4, 6 and 8 hours. Total RNA was extracted by TRIzol reagent as previously described. The level of SLC31A1 mRNA was measured by qRT‐PCR, and the half‐life of mRNA was obtained by non‐linear regression analysis.

### 2.12 Luciferase reporter assay

The human SLC31A1 3′‐UTR luciferase reporter vector and mutant SLC31A1 3′‐UTR vector containing a mutation in the predicted PTBP1 binding sequence (CUCUCU to AAAAA) were purchased from Genechem. Briefly, cells were seeded in 6‐well plates, transfected with Renilla luciferase vector and pGL3 reporter for 48 hours, and the luciferase activity was measured by a Dual‐Luciferase Reporter Assay System (Promega).

### 2.13 Measurement of intracellular Pt accumulation

The level of intracellular Pt accumulation was detected by inductively coupled plasma mass spectrometry (ICP‐MS) and performed by the toxicology laboratory of the School of Public Health, Tongji Medical College. The detection values were standardized by protein concentrations, which were determined by a BCA Protein Assay kit (Beyotime).

### 2.14 Establishment of in vivo cisplatin‐resistant osteosarcoma xenograft model

Twelve 4‐week‐old female nude mice were randomly divided into two groups: group A (8 mice) and group B (4 mice). In group A, 5 × 10^6^ MG‐63_CISR_ cells were injected subcutaneously into the right hind flanks of nude mice. Similarly, 5 × 10^6^ MG‐63_CISR_ cells with stably PTBP1 knock‐down were injected subcutaneously into the right hind flanks of nude mice in group B. Two weeks later, when the tumour volume reached approximately 100 mm^3^, group A was randomly divided into two groups on average, one group was given 2 mg/kg of cisplatin per day, the other group was given the same volume of saline, and the group B was given 2 mg/kg of cisplatin per day. Tumour volume was measured with a vernier calliper on 7, 14 and 21 days. All animals were killed on 21 days, and the mass of tumours was weighed.

The mice were obtained from Charles River Laboratory, and all animal care and experiments were conducted according to the guidelines of the Ethics Committee of Animal Experiment of Tongji Medical College.

### 2.15 TUNEL assay

Formalin‐fixed osteosarcoma tissues were embedded in paraffin and cut into 4‐μm sections. The TUNEL assay was performed with the One Step TUNEL Apoptosis Assay kit (Beyotime). Briefly, the paraffin‐embedded sections were dewaxed and washed for 20 minutes with 20 μg/mL DNase‐free protease K at 37°C and then washed three times with PBS. The TUNEL assay solution was allocated according to the instructions, and sections were incubated in TUNEL assay solution at 37°C for 60 minutes in the dark. After PBS washed three times, the nuclei were stained with DAPI. Fluorescence images were captured by the fluorescence microscopy (Olympus).

### 2.16 HE staining and immunohistochemical staining

For haematoxylin and eosin (HE) staining, the sections were stained with haematoxylin aqueous solution for 10 minutes, rinsed with running water for 1 hour, dehydrated in 70% and 90% alcohol and finally stained with eosin dye solution for 10 minutes. For immunohistochemical staining, the sections were blocked with 5% BSA and then stained with an anti‐PTBP1 antibody (1:100), anti‐SLC31A1 antibody (1:100), anti‐Cleaved Parp antibody (1:100), anti‐Cleaved Caspase‐3 antibody (1:100), anti‐Bax antibody (1:100) or anti‐Bcl‐2 antibody (1:100) at 4°C overnight. Finally, the section was incubated with an HRP‐labelled goat anti‐rabbit secondary antibody. Tissue sections were scanned by Pannoramic 250 Flash III (3DHistech Ltd.). Image acquisition and analysis were completed by CaseViewer software (3DHistech Inc) and Image‐Pro Plus software (Media Cybernetics Inc).

### 2.17 Statistical analysis

All experiments were independently repeated at least three times. SPSS 22.0 software and GraphPad Prism 7.0 software were used for all statistical analyses in this study. The difference between groups was analysed by 2‐tailed Student's *t* test or ANOVA. Data are presented as the mean ± SD. A *P* value < .05 was considered as statistically significant.

## 3 RESULTS

### 3.1 PTBP1 expression is up‐regulated in several common cancers

To explore the expression level of PTBP1 in several common cancers, we initially conducted bioinformatics analysis of the TCGA data set. We found that PTBP1 expression was increased in several common cancers, including BLCA, COAD, KIRC, LIHC, LUAD and STAD (Figure S1A‐C,G‐I). However, only BLCA and LIHC cohorts showed a separation between the normal and cancer samples (Figure S1A,G), and the expression of PTBP1 in the normal samples falls within the 10%‐90% range of the cancer cohort in other cancer types (Figure S1B,C,H‐I). For overall survival rate, in KIRC and LUAD, although high PTBP1 drives poorer outcome (Figure S1F,K), the expression level of PTBP1 in normal tissues is very close to that in cancer tissues. In the other four cancers, PTBP1 expression did not affect the prognosis (Figure S1D,E,J,L). These results suggest that although PTBP1 expression exhibits an increasing tendency in some human cancers, the relationship between PTBP1 level and prognosis is uncertain.

### 3.2 PTBP1 up‐regulation is associated with poor response to chemotherapy and serves as a prognostic factor in osteosarcoma

**Figure 1:**
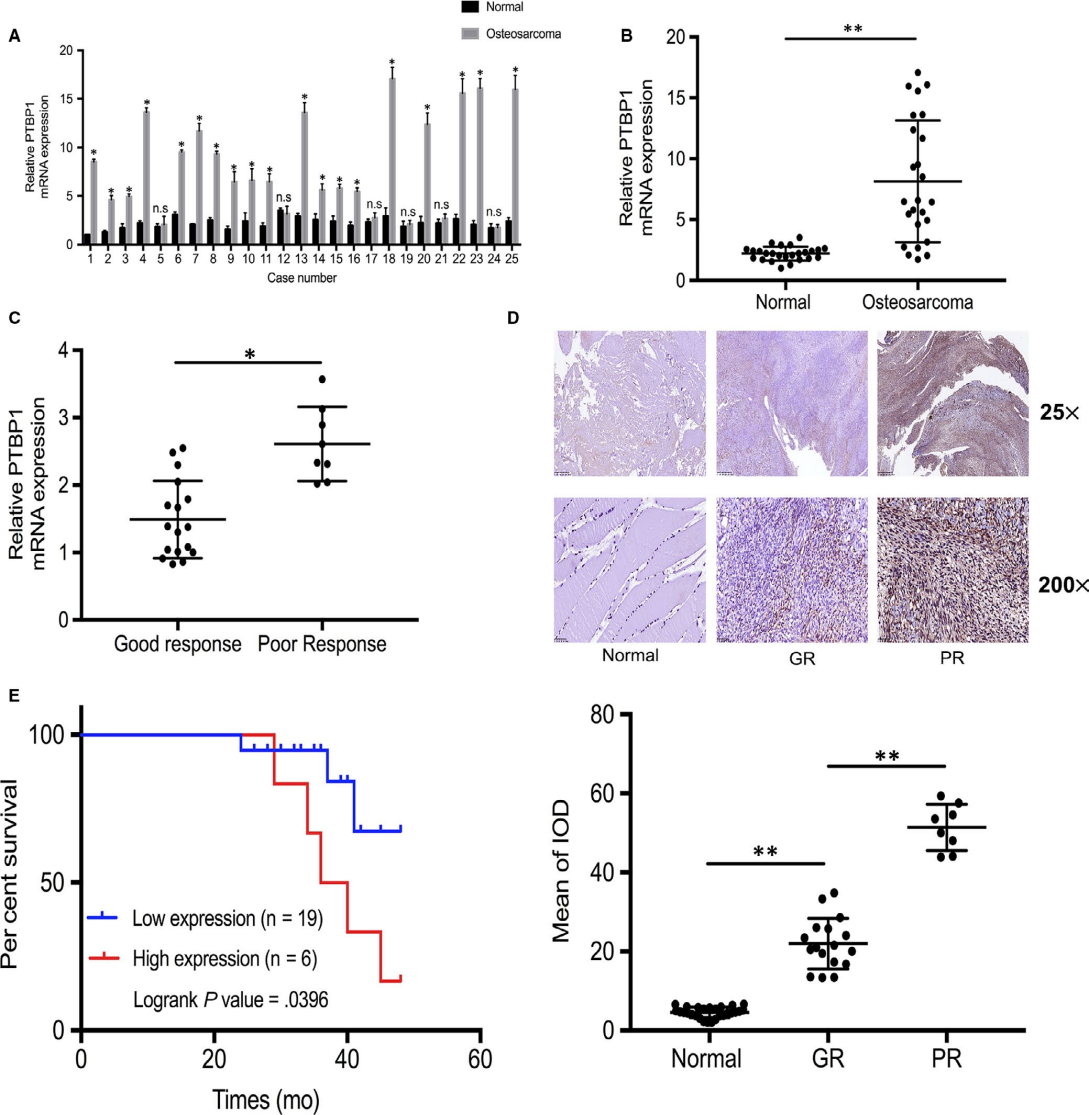
PTBP1 up‐regulation is associated with poor response to chemotherapy and serves as a prognostic factor in osteosarcoma. A and B, Detection of PTBP1 expression in 25 pairs of osteosarcoma tissues and matched normal tissues by real‐time quantitative polymerase chain reaction (qRT‐PCR). C, In cases with poor response (PR) to chemotherapy and cases with good response (GR) to chemotherapy, PTBP1 mRNA level was measured by qRT‐PCR. D, PTBP1 expression in normal tissues, GR tissues and PR tissues was measured by immunohistochemistry (IHC). Scale bars: 500 μm (25×) and 50 μm (200×). IOD is integrated optical density. E, Kaplan‐Meier plot survival time in patients with low and high PTBP1 expression. *P < .05, **P < .01

Because PTBP1 expression exhibits an increasing tendency in some common cancers, we investigated whether PTBP1 has a role in osteosarcoma, especially in the chemoresistance of osteosarcoma. We first assessed the expression of PTPB1 in 25 pairs of osteosarcoma tissues and adjacent normal tissues by qRT‐PCR. As shown in Figure 1A,B, the PTBP1 expression level was higher in 19 osteosarcoma tissues than that in the matched normal tissues. In the remaining 6 cases, there was no significant difference in PTBP1 levels between tumours and normal tissues. According to the pathological diagnosis, 8 osteosarcoma patients were classified as having a poor response to chemotherapy, and 17 patients were defined as having a good response. Then, qRT‐PCR and immunohistochemistry confirmed that the expression level of PTBP1 was higher in poor responders than in good responders (Figure 1C,D). Moreover, we processed the clinical data of all patients to explore the relationship between PTBP1 expression and the clinical and pathological characteristics in 25 osteosarcoma patients. The results showed that the expression of PTBP1 was not correlated with age, gender, location of tumorigenesis, pathological classification and pathological grading of tumours (Table 1). Additionally, Kaplan‐Meier analysis revealed that patients with high expression levels of PTBP1 had a poor overall survival rate (Figure 1E). Taken together, these results demonstrate that PTBP1 up‐regulation is associated with poor response to chemotherapy of osteosarcoma, and higher PTBP1 expression predicts worse prognoses in osteosarcoma patients.

**Table 1:** Characteristics of patients with osteosarcoma

Variables	PTBP1 low (n = 19)	PTBP1 high (n = 6)	*P* value*
Gender			
Male	11 (57.89%)	3 (50%)	.734
Female	8 (42.11%)	3 (50%)	
Age (y)			
Median	15.3	14.6	>.99
Range	8‐19	6‐21	
Anatomical site			
Femur	6 (31.58%)	2 (33.33%)	.840
Tibia	6 (31.58%)	2 (33.33%)	
Humerus	4 (21.05%)	1 (16.67%)	
Pelvis	2 (10.53%)	0 (0.00%)	
Other	1 (5.26%)	1 (16.67%)	
Histologic subtype			
Osteoblastic	9 (47.37%)	3 (50.00%)	.691
Chondroblastic	4 (21.05%)	2 (33.33%)	
Fibroblastic	4 (21.05%)	1 (16.67%)	
Telangiectatic	1 (5.26%)	0 (0.00%)	
Other	1 (5.26%)	0 (0.00%)	
Histologic grade			
III	7 (36.84%)	2 (33.33%)	.876
IV	12 (63.16%)	4 (66.67%)	
Enneking grade			
IIA	8 (42.11%)	3 (50%)	.734
IIB	11 (57.89%)	3 (50%)	

* The difference in variables between the two groups was measured by chi‐square test.

### 3.3 PTBP1 is up‐regulated in osteosarcoma cell lines and cisplatin‐resistant cell lines

Aberrant expression of PTBP1 has been observed in osteosarcoma tissues, and it is associated with poor response to chemotherapy. Next, we examined the level of PTBP1 in osteosarcoma cells and CISR osteosarcoma cells. Real‐time quantitative PCR and Western blot analysis showed that the expression of PTBP1 in the osteosarcoma cell lines MG‐63 and U‐2OS was higher than that in the human osteoblast cell line NHOst (Figure S2A,B). In the CISR cell lines MG‐63_CISR_ and U‐2OS_CISR_, the expression intensity of PTBP1 was stronger than that in MG‐63 and U‐2OS cell lines (Figure S2C‐F). Together, these results suggest that PTBP1 up‐regulation is associated with the cisplatin resistance of osteosarcoma at the cellular level.

### 3.4 PTBP1 deficiency enhances cisplatin efficacy in CISR osteosarcoma cells

**Figure 2:**
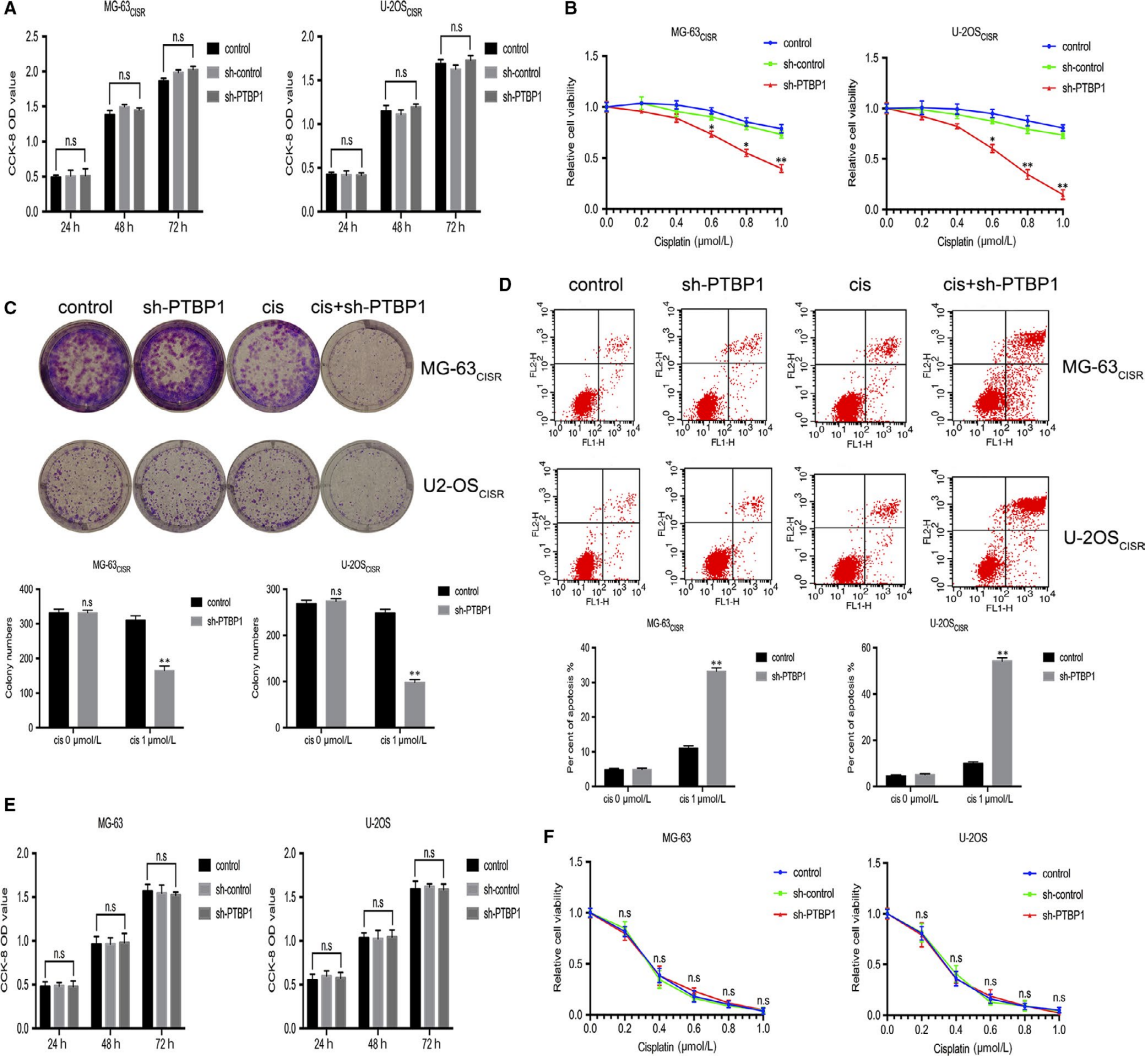
PTBP1 deficiency enhances cisplatin efficacy in CISR osteosarcoma cells. A, The effect of PTBP1 knock‐down on cell viability of CISR osteosarcoma cells was measured by CCK‐8 assay. B, The effect of PTBP1 knock‐down on cisplatin‐induced inhibition of cell viability of CISR osteosarcoma cells was verified by CCK‐8 assay. C, Colony formation assay examined the effects of PTBP1 knock‐down, cisplatin alone and PTBP1 knock‐down combined with cisplatin on colony formation ability of CISR osteosarcoma cells. D, Flow cytometric analysis was used to detect the effects of PTBP1 knock‐down, cisplatin alone and PTBP1 knock‐down combined with cisplatin on cell apoptosis of CISR osteosarcoma cells. E, The effect of sh‐PTBP1 on cell viability of MG‐63 and U‐2OS cells was measured by CCK‐8 assay. F, The effect of sh‐PTBP1 on cisplatin‐induced inhibition of cell viability of MG‐63 and U‐2OS cells was verified by CCK‐8 assay. *P < .05, **P < .01, n.s is no significance

To further investigate the role of PTBP1 in CISR osteosarcoma cells, we silenced PTBP1 expression in MG‐63_CISR_ and U‐2OS_CISR_ cells using shRNA specific for PTBP1. First, we validated the transfection efficiency of sh‐PTBP1 in CISR cells by qRT‐PCR and Western blot (Figure S3A,B). According to the results, we chose sh‐PTBP1 #1 for subsequent experiments. The CCK‐8 assay and colony formation assay were used to detect the sensitivity of MG‐63_CISR_ and U‐2OS_CISR_ cells to cisplatin after PTBP1 silenced. The results indicated that PTBP1 knock‐down alone had no obviously effect on cell viability and colony formation ability but enhanced the effect of cisplatin on CSIR cells (Figure 2A‐C). In addition, cell apoptosis was not affected by the suppression of PTBP1, whereas the number of apoptotic cells induced by cisplatin increased significantly upon PTBP1 knock‐down (Figure 2D). To determine whether the effect of sh‐PTBP1 on cisplatin sensitivity is specific to the CISR cells, we transfected MG‐63 and U‐2OS cells with sh‐PTBP1 and performed CCK‐8 assay. We found that sh‐PTBP1 has no significant effect on cell viability and cisplatin sensitivity of MG‐63 and U‐2OS cells (Figure 2E,F). Overall, these results reveal that inhibiting PTBP1 expression could enhance the killing effect of cisplatin on CISR osteosarcoma cells.

### 3.5 PTBP1 interacts with SLC31A1 mRNA in CISR osteosarcoma cells

**Figure 3:**
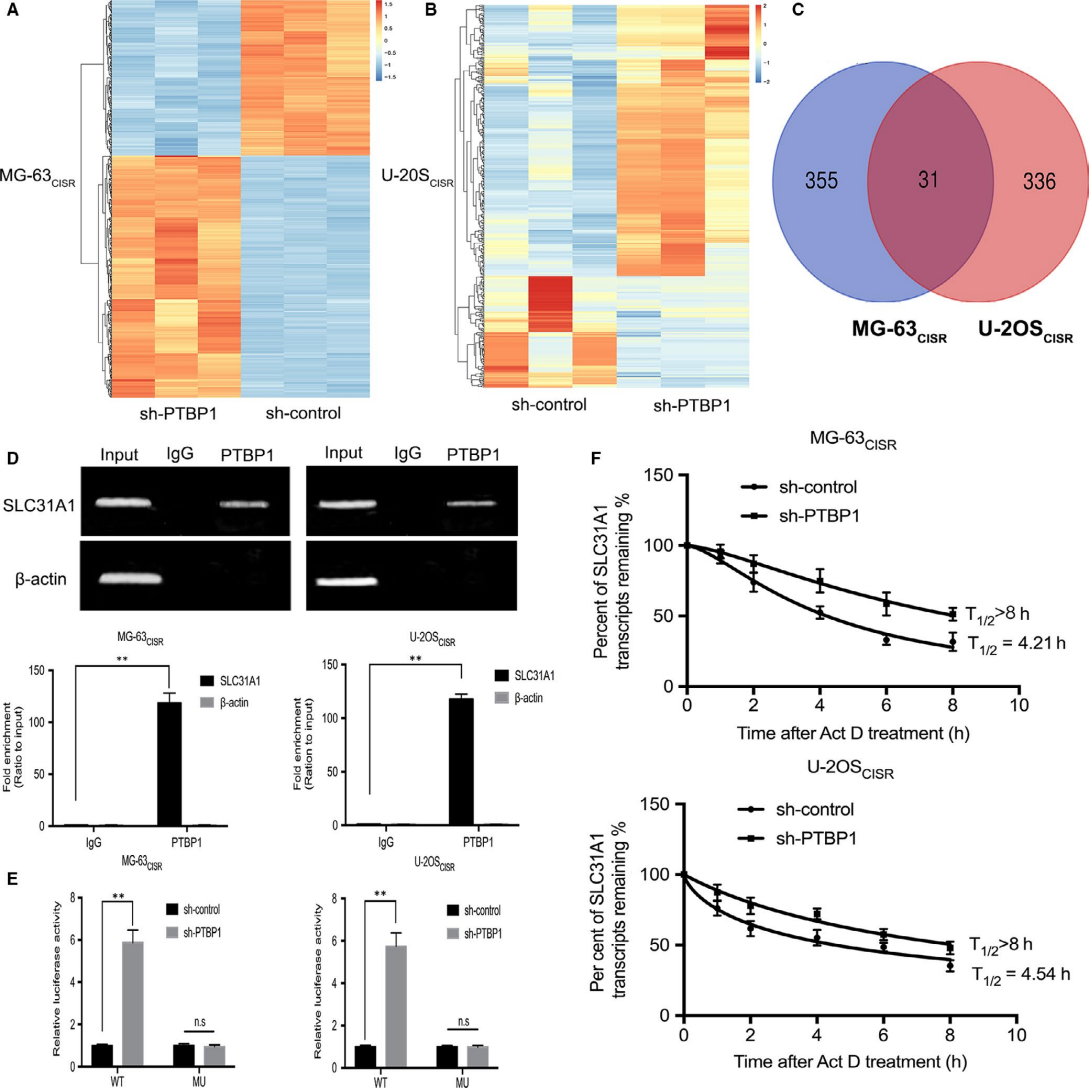
PTBP1 regulates the expression of SLC31A1 in CISR osteosarcoma cells. A and B, Transcriptome sequencing was performed after knocking down PTBP1 in MG‐63_CISR_ and U‐2OS_CISR_ cells. Target genes which fold change > 2 and FDR < 0.05 were selected for cluster analysis and represent as heat maps. Blue indicates down‐regulation of gene expression, and red indicates up‐regulation of gene expression. C, Venn diagram shows the overlap of highly expressed genes in two sets of sequencing results. D, RNA immunoprecipitation in MG‐63_CISR_ and U‐2OS_CISR_ cells using an anti‐IgG control or anti‐PTBP1 antibody. SLC31A1 and β‐actin mRNA abundance in immune‐precipitated fraction was measured by qRT‐PCR. E, The SLC31A1 3′‐UTR reporter constructs containing the wild‐type (WT) or mutant (MU) PTBP1 binding sequences were co‐transfected with sh‐control or sh‐PTBP1 into MG‐63_CISR_ and U‐2OS_CISR_ cells and luciferase activity levels measured. F, The relative level of SLC31A1 mRNA after treated with 5 μg/mL actinomycin D for 0, 2, 4, 6 and 8 h was measured by qRT‐PCR, and the half‐life of SLC31A1 mRNA was analysed by non‐linear regression analysis. **P < .01, n.s no significance

Since PTBP1 is a typical RNA‐binding protein, the post‐transcriptional regulation of target genes is likely to be a potential molecular mechanism for its function. To identify the downstream targets of PTBP1, we performed transcriptome sequencing using total RNA from PTBP1 knock‐down MG‐63_CISR_ and U‐2OS_CISR_ cells. With cut‐off criteria (|log_2_FoldChange| ≥ 1 and *P* value < .05), the results exhibited that the expression of 386 genes was up‐regulated and 245 genes was down‐regulated in PTBP1 knock‐down MG‐63_CISR_ cells compared with the control MG‐63_CISR_ cells (Figure 3A). Moreover, the expression of 367 genes was up‐regulated and 151 genes was down‐regulated in PTBP1 knock‐down U‐2OS_CISR_ cells compared with the control U‐2OS_CISR_ cells (Figure 3B). All differentially expressed genes were listed in Tables S1 and S2 and subsequently performed Gene Ontology (GO) analysis (Figure S4A,B) and KEGG Pathway analysis (Figure S4C,D). According to previous studies on PTBP1 in cancer, we found that PTBP1 could reduce the stability of mRNA by binding to it in a variety of cancer cells, which further negatively regulates the expression of the target gene.^28^, ^30^, ^34^ Therefore, we hypothesized that PTBP1 functions by reducing the stability of downstream target RNA and inhibiting the expression of them. Through analysis of the data, we found that 31 genes overlapped within up‐regulated expression group, and among them, the expression of SLC31A1 was most significantly affected by PTBP1 (the *P* value was the lowest) (Figure 3C). To further verify whether PTBP1 directly binds to SLC31A1 mRNA, RNA immunoprecipitation assay was performed in MG‐63_CISR_ and U‐2OS_CISR_ cells. The results confirmed that SLC31A1 mRNA was significantly enriched in anti‐PTBP1 antibody co‐precipitated RNA fragments (Figure 3D), suggesting that PTBP1 binds to SLC31A1 mRNA in these cells. The results of the dual‐luciferase reporter assay showed that compared with the sh‐control, we detect a significant increase in luciferase activity of wild‐type SLC31A1 3′‐UTR reporter when transfected with sh‐PTBP1 in CISR cells. However, down‐regulation of PTBP1 had no repressive effect on the mutant SLC31A1 3′‐UTR construct (Figure 3E). Thereafter, we carried out the RNA stability assay to analysis the half‐life of SLC31A1 mRNA. As shown in Figure 3F, the half‐life of SLC31A1 mRNA was 4.21 and 4.54 hours in MG‐63_CISR_ and U‐2OS_CISR_ cells, respectively, while the half‐life of SLC31A1 was longer than 8 hours in PTBP1 knock‐down cells. Taken together, these results indicate that PTBP1 can bind to the messenger RNA of SLC31A1, which further accelerates its degradation.

### 3.6 SLC31A1 is down‐regulated in chemotherapy‐insensitive osteosarcoma tissues and CISR osteosarcoma cells

**Figure 4:**
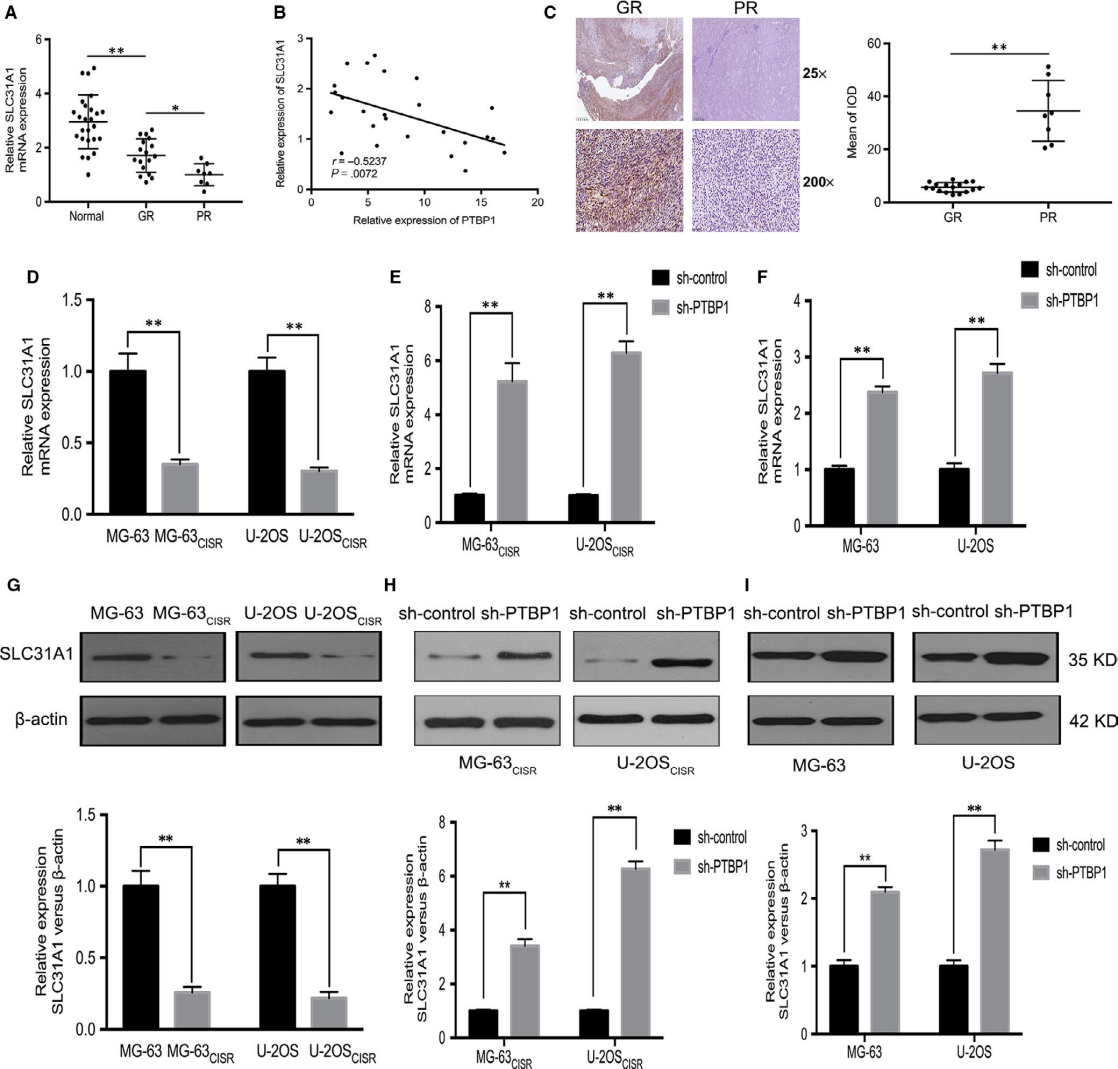
SLC31A1 is down‐regulated in chemotherapy‐insensitive osteosarcoma tissues and CISR osteosarcoma cells. A, The level of SLC31A1 mRNA in osteosarcoma tissues was measured by qRT‐PCR. B, Linear regression analysis of the correlation between the expression levels of SLC31A1 and PTBP1 in osteosarcoma. C, SLC31A1 expression in GR tissues and PR tissues was measured by IHC. Scale bars: 500 μm (25×) and 50 μm (200×). D and G, The expression of SLC31A1 in MG‐63, U‐2OS and their cisplatin‐resistant sub‐lines was measured by qRT‐PCR and Western blot. E and H, The expression of SLC31A1 in MG‐63_CISR_, U‐2OS_CISR_ cells and PTBP1 knock‐down MG‐63_CISR_, U‐2OS_CISR_ cells was measured by qRT‐PCR and Western blot. F and I, The expression of SLC31A1 in MG‐63, U‐2OS cells and PTBP1 knock‐down MG‐63, U‐2OS cells was measured by qRT‐PCR and Western blot. *P < .05, **P < .01

To further explore the role of SLC31A1 in osteosarcoma, we assessed the level of SLC31A1 in osteosarcoma tissues and CISR osteosarcoma cells. The results of qRT‐PCR analysis showed that the expression of SLC31A1 mRNA was down‐regulated in osteosarcoma tissues, especially lower in chemotherapy‐insensitive osteosarcoma tissues, and there was a negative correlation between the expression of SLC31A1 and PTBP1 in osteosarcoma tissue (Figure 4A,B). Moreover, IHC staining showed that the positive rate of SLC31A1 in good responders was significantly higher than that in poor responders (Figure 4C). qRT‐PCR and Western blot analysis demonstrated that the levels of SLC31A1 in MG‐63_CISR_ and U‐2OS_CISR_ cells were significantly lower than that in their original cells (Figure 4D,G). Moreover, PTBP1 knock‐down increased the expression level of SLC31A1 in MG‐63_CISR_ and U‐2OS_CISR_ cells (Figure 4E,H). To determine whether sh‐PTBP1 in MG‐63 and U‐2OS cells has a similar effect on increasing SLC31A1 expression, we transfected MG‐63 and U‐2OS cells with sh‐PTBP1 and performed qRT‐PCR and Western blot assay to measure the level of SLC31A1 in PTBP1 knock‐down cells. The results implied that PTBP1 knock‐down in cisplatin‐sensitive osteosarcoma cells also increased the expression of SLC31A1 (Figure 4F,I). Together, these results suggest that the expression of SLC31A1 is decreased in chemotherapy‐insensitive osteosarcoma tissues and CISR osteosarcoma cells, and the effect of PTBP1 knock‐down on SLC31A1 up‐regulation is not unique to CISR cells.

### 3.7 SLC31A1 knock‐down abrogates the effect of PTBP1 silence on the chemosensitivity of CISR osteosarcoma cells

**Figure 5:**
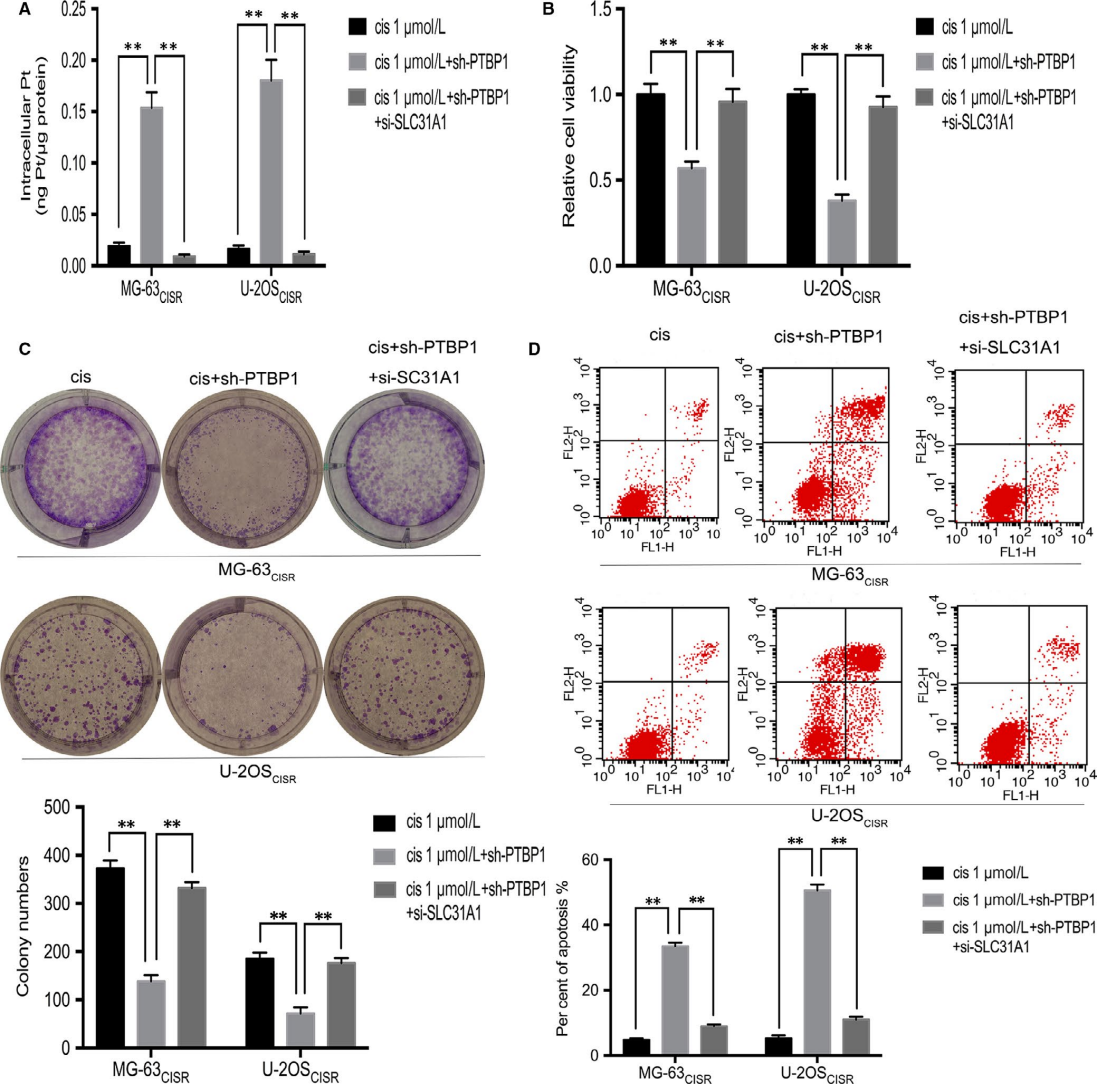
SLC31A1 knock‐down abrogates the effect of PTBP1 silence on the chemosensitivity of CISR osteosarcoma cells. A, In MG‐63_CISR_ and U‐2OS_CISR_ cells, intracellular platinum concentration was determined by inductively coupled plasma mass spectrometry (ICP‐MS). B and C, CCK‐8 assay and colony formation assay determined the cell viability and colony formation ability of MG‐63_CISR_ and U‐2OS_CISR_ cells. D, Flow cytometric analysis of apoptosis was used to detect the effects of cisplatin alone. PTBP1 knock‐down combined with cisplatin, PTBP1 knock‐down combined with cisplatin simultaneously inhibited SLC31A1 on cell apoptosis. **P < .01

To evaluate whether SLC31A1 mediates PTBP1 functions, we carried out rescue experiments by knocking down SLC31A1 in CISR cells. Initially, qRT‐PCR and Western blot were used to validate the transfection efficiency of the SLC31A1 siRNA in MG‐63_CISR_ and U‐2OS_CISR_ cells (Figure S3C,D). Intracellular platinum concentration measurements showed that PTBP1 knock‐down resulted in intracellular platinum accumulation, and after the inhibition of SLC31A1, the intracellular platinum concentration decreased accordingly (Figure 5A). The cell viability assay and colony formation assay results indicated that that the inhibition of SLC31A1 abrogated the effects of PTBP1 knock‐down on cell sensitivity to cisplatin (Figure 5B,C). In addition, the percentage of cells that underwent apoptosis was decreased significantly after SLC31A1 knock‐down (Figure 5D). As expect, these data suggest that SLC31A1 mediates the effect of PTBP1 knock‐down on the chemosensitivity of CISR osteosarcoma cells by regulating the uptake of cisplatin.

### 3.8 SLC31A1 mediates the regulation of PTPB1 on cisplatin‐induced apoptosis‐related genes expression

**Figure 6:**
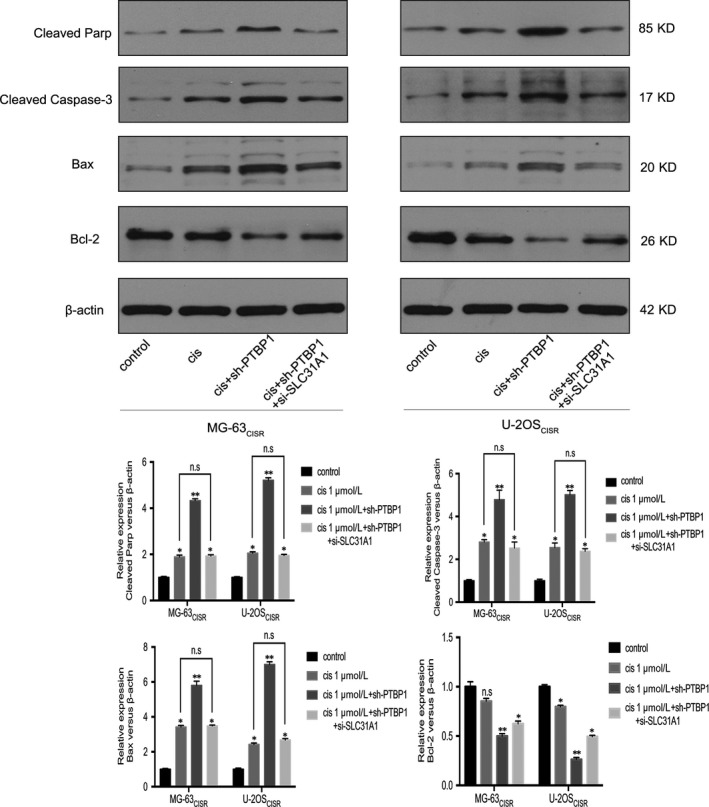
SLC31A1 mediates the regulation of PTPB1 on cisplatin‐induced apoptosis‐related genes expression. Western blot assay was used to measure the protein expression of pro‐apoptotic related genes: Cleaved Parp, Cleaved Caspase‐3, Bax, and anti‐apoptotic gene Bcl‐2 after different treatments. *P < .05, **P < .01, n.s is no significance

The mitochondrial pathway is one of the main ways of cisplatin‐induced cancer cell apoptosis.^5^, ^9^ As mentioned above, PTBP1 knock‐down results in the up‐regulation of SLC31A1 and the accumulation of intracellular cisplatin. Therefore, we hypothesized that PTBP1 and SLC31A1 could affect the expression of genes related to the mitochondrial apoptotic pathway. To validate our assumption, we examined the expression of apoptosis‐related genes in mitochondrial pathway in two kinds of CISR cells by Western blot. As shown in Figure 6, the levels of the pro‐apoptotic genes Cleaved Parp, Cleaved Caspase‐3 and Bax were up‐regulated, and the level of the anti‐apoptotic gene Bcl‐2 did not change after 48 hours of intervention with 1 μmol/L cisplatin alone. After 48 hours of treatment with 1 μmol/L cisplatin in PTBP1 knock‐down cells, the expression of these pro‐apoptotic genes increased significantly, and the expression of Bcl‐2 decreased significantly. However, SLC31A1 silencing reversed the effect of PTBP1 on cisplatin‐induced pro‐apoptotic genes expression, and the expression of anti‐apoptotic genes remained at a relatively low level. Taken together, SLC31A1 mediates the effect of PTPB1 on the expression of apoptosis‐related genes induced by cisplatin.

### 3.9 PTBP1 knock‐down potentiates the anti‐tumour effect of cisplatin on cisplatin‐resistant osteosarcoma in vivo

**Figure 7:**
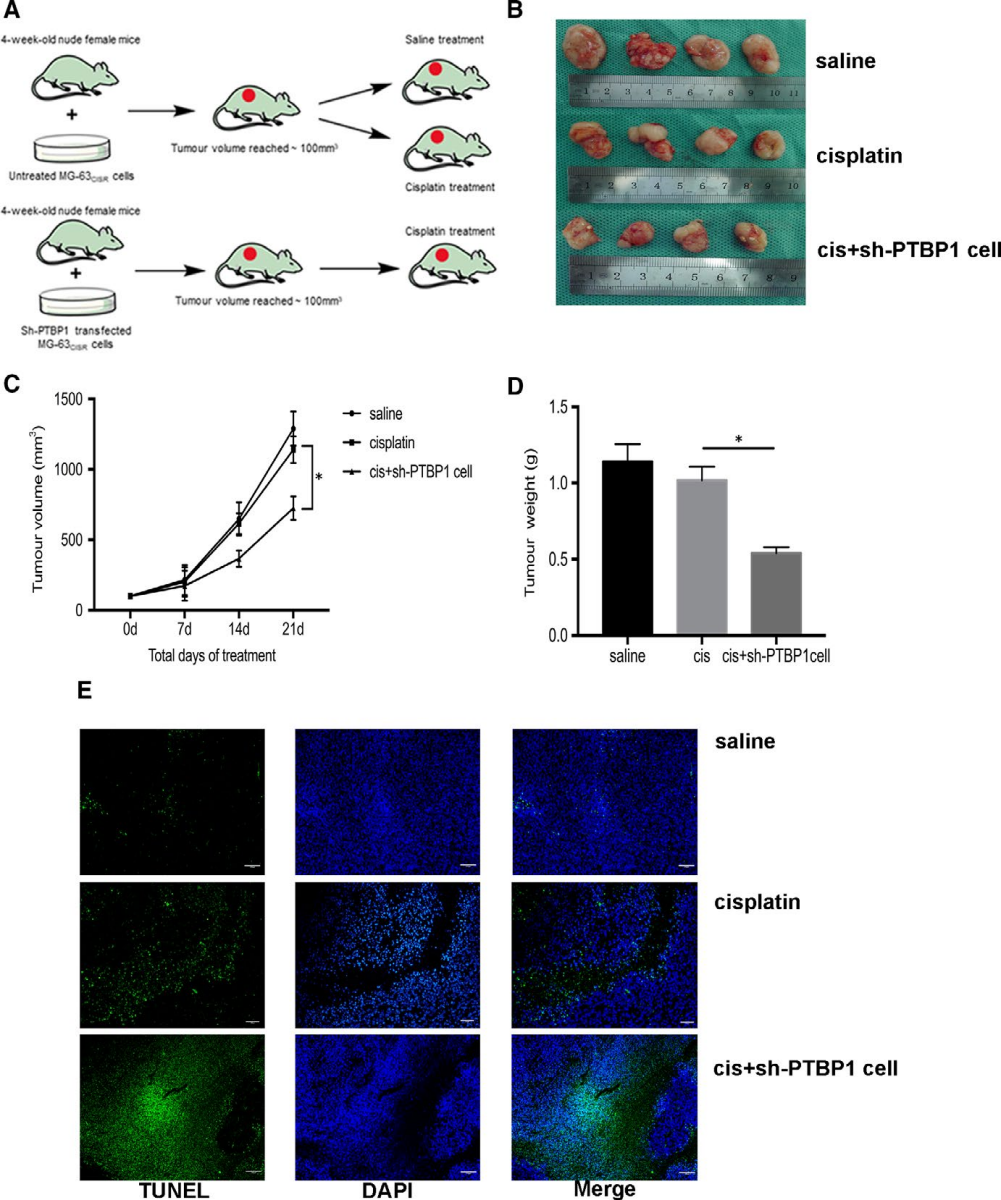
PTBP1 knock‐down potentiates the anti‐tumour effect of cisplatin on cisplatin‐resistant osteosarcoma in vivo. A, The treatment scheme. B, The general view of xenograft tumours in nude mice after 21 d of different treatments. C, Volume of xenograft tumours after 0, 7, 14 and 21 d of different treatments. D, Mass of xenograft tumours after 21 d of different treatments. E, Tunel assay determined the killing effect of different treatments on xenograft tumours in nude mice. Scale bar: 200 μm (40×). *P < .05

**Figure 8:**
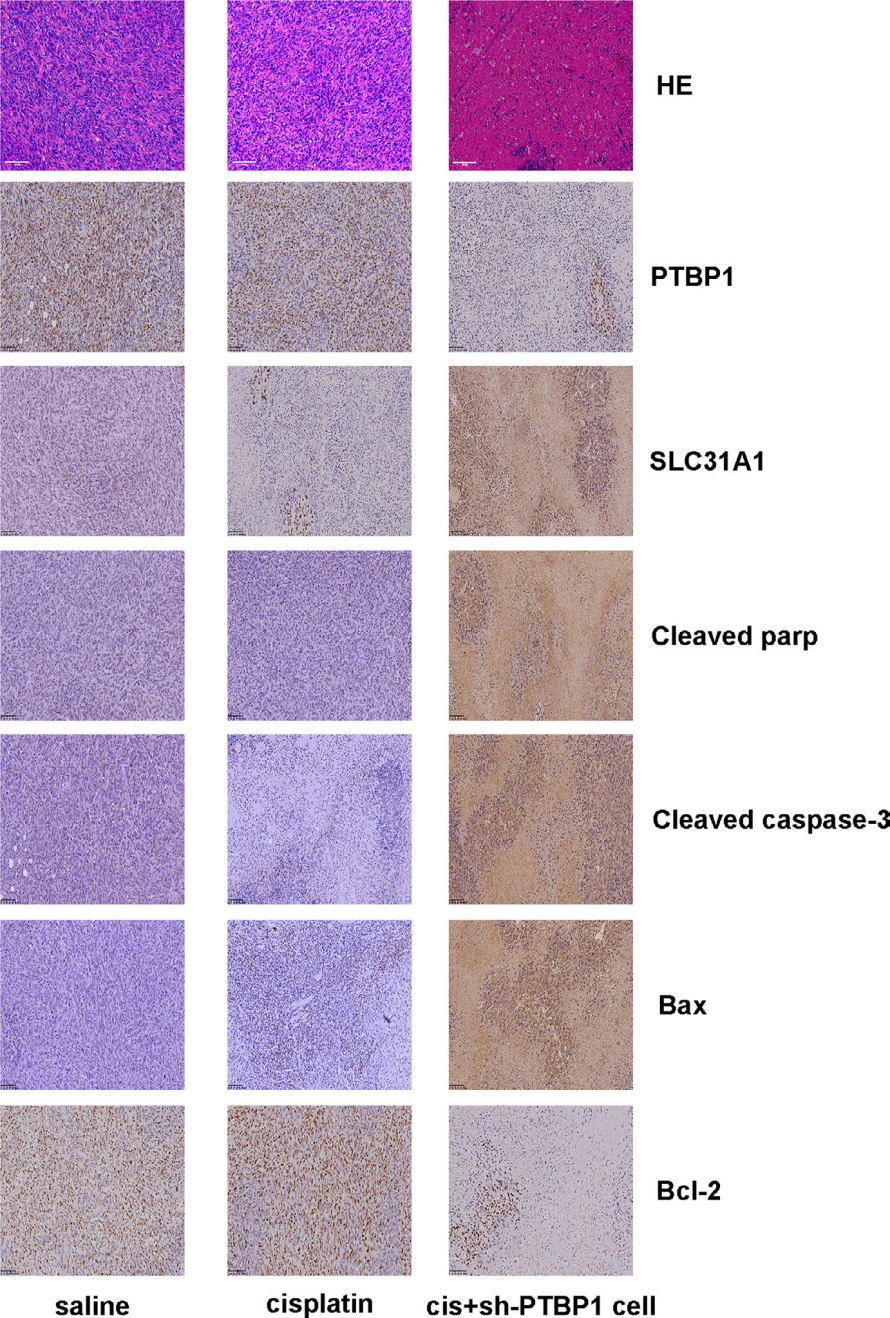
Haematoxylin and eosin (HE) and IHC staining of xenograft osteosarcoma. HE staining and IHC staining were used to detect the nucleus fragmentation and the expression of related genes in xenograft tumours. Scale bar: 100 μm (100×)

Through a series of experiments in vitro, we found that PTBP1 knock‐down could overcome the cisplatin resistance of MG‐63_CISR_ and U‐2OS_CISR_ cells. To further investigate the effect of PTBP1 knock‐down in vivo, we carried out osteosarcoma xenograft and treatment experiments in nude mice. The treatment scheme is shown in Figure 7A. The results revealed that treatment with 2 mg/kg cisplatin alone had no obvious effects on CISR osteosarcoma in vivo. However, cisplatin could significantly inhibit the growth of PTBP1 knock‐down osteosarcoma xenograft in vivo (Figure 7B‐D). TUNEL assays showed that treatment with cisplatin alone caused only minimal osteosarcoma apoptosis. In contrast, cisplatin treatment combined with PTBP1 knock‐down induced apoptosis in large areas of tumours (Figure 7E). In the HE staining, we found that the combination of PTBP1 knock‐down and cisplatin treatment resulted in increased nuclear fragmentation. Moreover, immunohistochemistry (IHC) staining indicated that PTBP1 knock‐down combined with cisplatin treatment produced a significant pro‐apoptotic effect in osteosarcoma in vivo (Figure 8). Together, PTBP1 knock‐down potentiates the anti‐tumour effect of cisplatin on cisplatin‐resistant osteosarcoma in vivo.

## 4 DISCUSSION

Since adjuvant chemotherapy has been widely used in the treatment of osteosarcoma, the prognosis of osteosarcoma patients has been greatly improved. However, one of the major barriers in curing osteosarcoma is the emergence of chemoresistance. Therefore, overcoming this obstacle is the main focus of the current research on osteosarcoma treatment. To solve the problem of chemoresistance in osteosarcoma, it is important to reveal the underlying molecular mechanism and to develop effective chemosensitizers for first‐line chemotherapeutic drugs. In this study, we demonstrated the biological roles of PTBP1 and SLC31A1 in chemoresistant osteosarcoma for the first time.

As one of the most critical participants in the regulation of intracellular RNA biological function, RNA‐binding proteins have been proved to be widely involved in the occurrence, progression and chemoresistance of various cancers.^23^, ^35^, ^36^ PTBP1 is a member of the heterogeneous nuclear ribonucleoprotein family as well as a typical RNA‐binding protein. In this study, we initially found that PTBP1 expression is increased in several common cancers, but it has no clear relationship with prognosis through bioinformatics analysis. On this basis, we revealed that PTBP1 is aberrantly expressed in osteosarcoma tissues and cells, especially those insensitive to chemotherapeutics. The survival analysis indicated that high expression of PTBP1 predicts the worse prognosis. Then, we conducted in vitro assays and in vivo experiments, and the results demonstrated that knock‐down PTBP1 in CISR osteosarcoma cells enhances the anti‐tumour effect of cisplatin. These findings suggest that PTBP1 acts as a determinant in regulating the chemosensitivity of osteosarcoma to cisplatin. Currently, a series of researches have demonstrated that PTBP1 is abnormally expressed in cancer tissues or cells and is associated with the pathological processes of cancer development.^37^, ^38^, ^39^ Most of the molecular mechanisms involved in these studies are post‐transcriptional regulation of PTBP1, which is determined by the nature of PTBP1 as an RNA‐binding protein.^40^, ^41^ Consequently, from the perspective of post‐transcriptional regulation, we identified SLC31A1 as the target of PTBP1 in CISR osteosarcoma cells by transcriptome sequencing, RIP assay and luciferase reporter assay.

Platinum drugs, like other essential metal ions, enter cells via the cell membranes and distribute, eventually being excreted extracellularly. The anti‐tumour efficiency of platinum chemotherapeutics depends on the concentration of platinum drugs in cancer cells,^5^, ^42^ while the vast majority of acquired chemoresistance is accompanied by a down‐regulation of intracellular platinum drugs aggregation.^43^ Previous studies have demonstrated that copper‐related transporters and metabolic systems play an important role in the intake, distribution and excretion of platinum.^44^, ^45^ The SLC31A1 gene encodes copper transporter 1 protein, which efficiently transport copper and maintain the homeostasis of copper in cells. A growing body of evidence suggests that SLC31A1 is responsible for the majority of the active transport of platinum drugs in cancer cells.^46^, ^47^ Abnormal expression of SLC31A1 in cancer cells affects the sensitivity of cells to platinum drugs and even directly leads to chemoresistance of cancer cells.^48^, ^49^ As above described, we identified SLC31A1 as the target of PTBP1 in CISR osteosarcoma cells. We further confirmed that SLC31A1 expression is lower in cisplatin‐insensitive osteosarcoma tissues and CISR cells. Furthermore, we validated the regulatory effect of PTBP1 on the expression of SLC31A1 and chemosensitivity enhanced by PTBP1 knock‐down is mediated by SLC31A1 in CISR osteosarcoma cells through a series of assays. It is noteworthy that PTBP1 knock‐down also up‐regulated the expression of SLC31A1 in MG‐63 and U‐2OS cells, but PTBP1 silencing did not enhance the response of these sensitive cell lines to cisplatin. We think the possible reason is that there are already high levels of SLC31A1 in MG‐63 and U‐2OS cells. These channels are sufficient for cells to take up external cisplatin. Besides, the cisplatin uptake by SLC31A1 accounts for 60%~70% of the total uptake.^17^ Therefore, even if the expression level of SLC31A1 is increased, more cisplatin cannot be taken up into cells when the external cisplatin concentration is unchanged. Of course, this requires our further verification and exploration.

In conclusion, our study reveals the role of PTBP1 in CISR osteosarcoma cells and its relevance with prognosis of osteosarcoma patients. PTBP1 affects the expression level of SLC31A1 by binding to its mRNA, thereby affecting the uptake of cisplatin by CISR osteosarcoma cells and regulating the sensitivity of CISR osteosarcoma cells to cisplatin. Our evidence provides a new perspective for the treatment of osteosarcoma chemoresistance.

## CONFLICT OF INTERESTS

All authors declare that they have no competing interests.

## Supporting information

Fig S1

Fig S2

Fig S3

Fig S4

Table S1

Table S2

Supplementary Material

## Data Availability

The data that support the findings of this study are available from the corresponding author upon reasonable request.
