# Supplementary figures and images for: PTBP1 modulates osteosarcoma chemoresistance to cisplatin by regulating the expression of the copper transporter SLC31A1
Source: J Cell Mol Med. 2020 Mar 24;24(9):5274–89. doi: 10.1111/jcmm.15183 (PMC7205786; doi:10.1111/jcmm.15183)

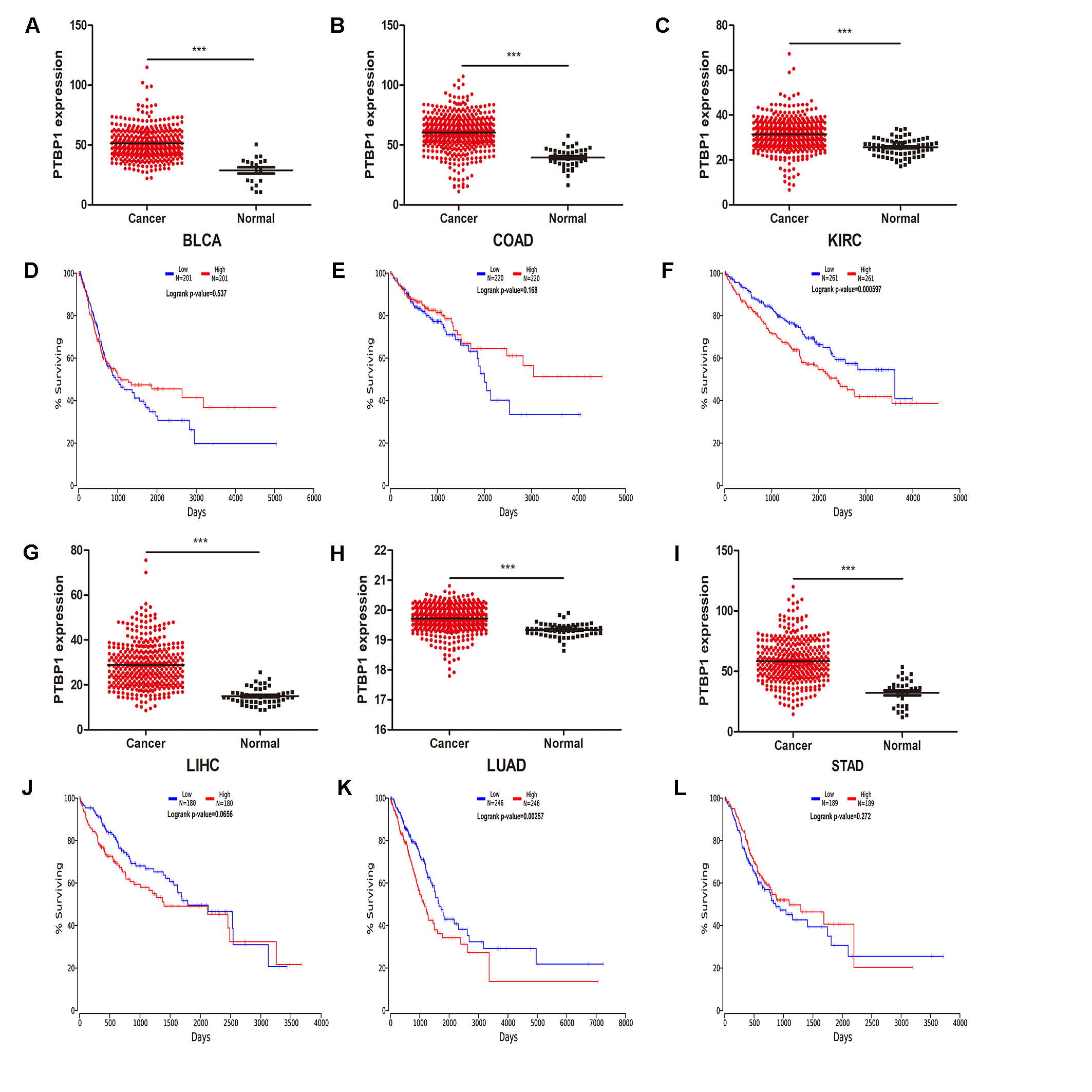

Supplement: Supplementary file 1 — Fig S1 [file JCMM-24-5274-s001.tif]

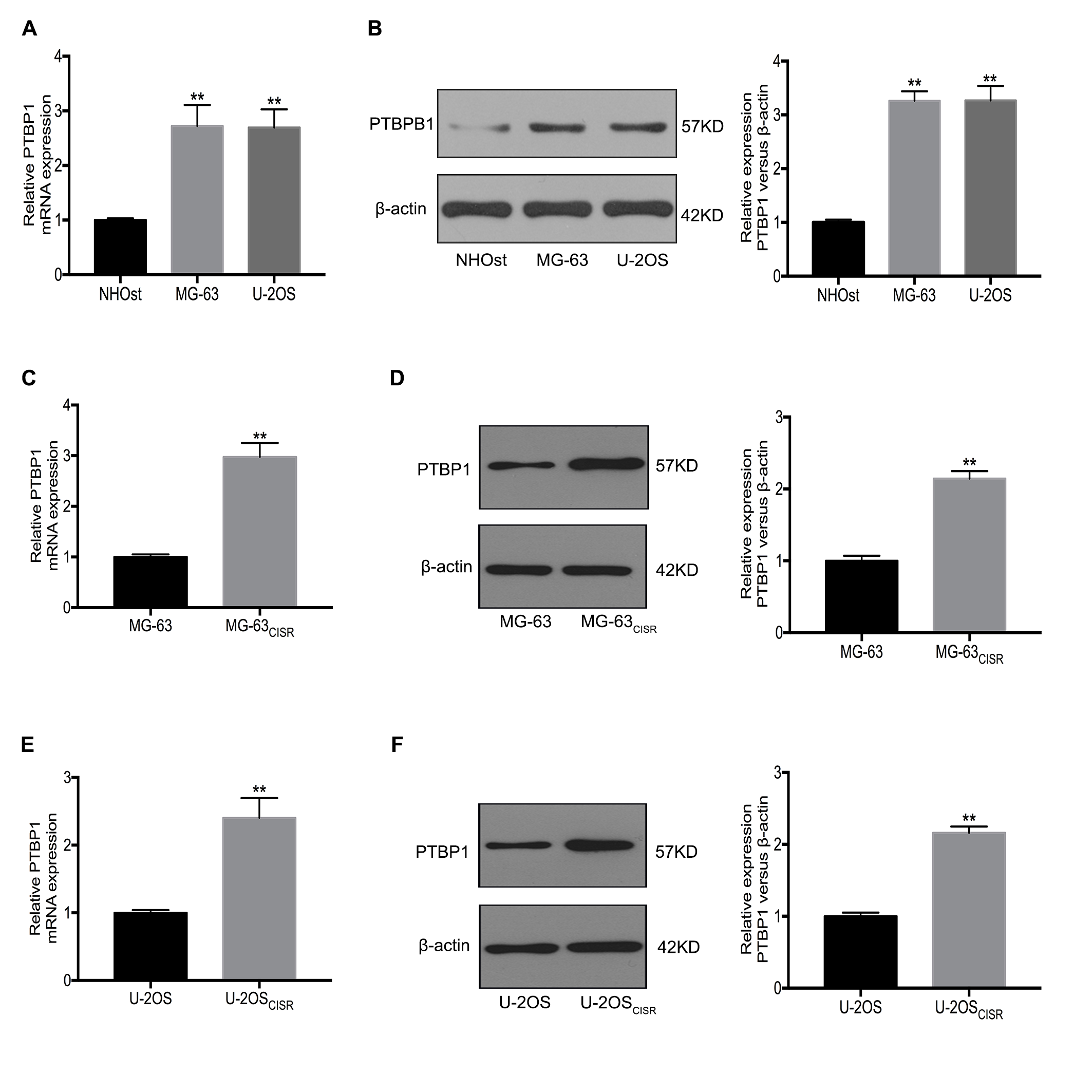

Supplement: Supplementary file 2 — Fig S2 [file JCMM-24-5274-s002.tif]

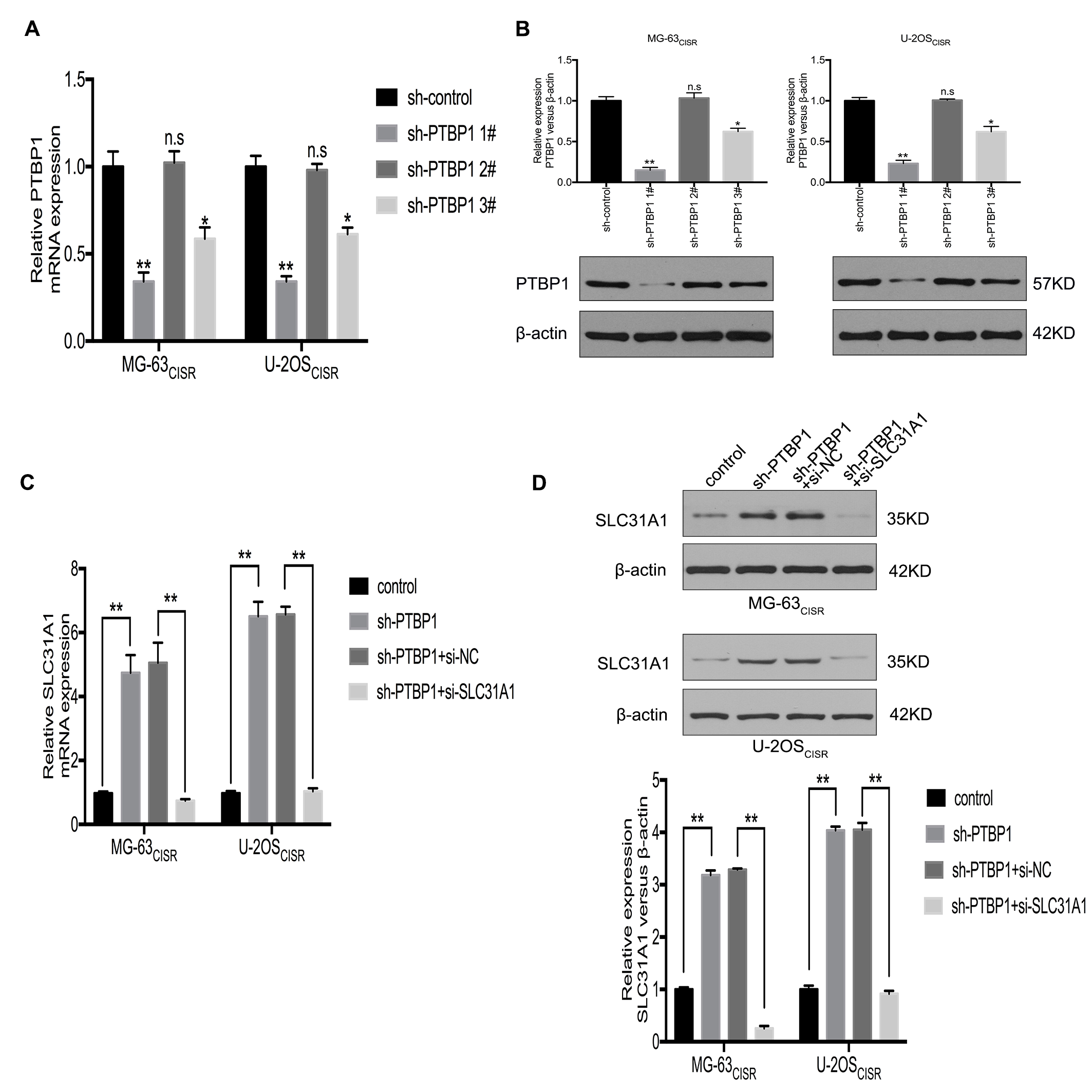

Supplement: Supplementary file 3 — Fig S3 [file JCMM-24-5274-s003.tif]

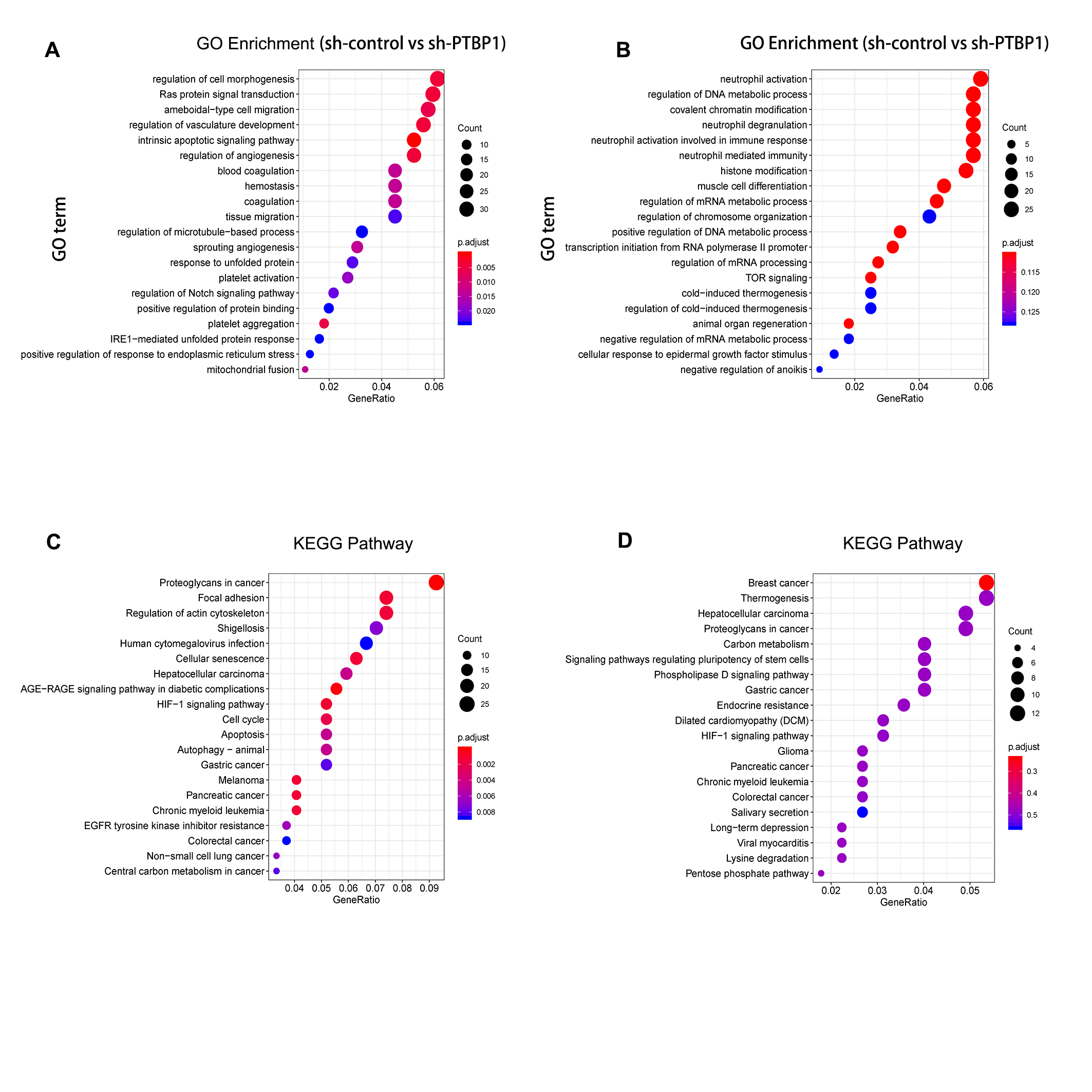

Supplement: Supplementary file 4 — Fig S4 [file JCMM-24-5274-s004.tif]
