# Supplementary material for: PTBP1 modulates osteosarcoma chemoresistance to cisplatin by regulating the expression of the copper transporter SLC31A1
Source: J Cell Mol Med. 2020 Mar 24;24(9):5274–89. doi: 10.1111/jcmm.15183 (PMC7205786; doi:10.1111/jcmm.15183)
Supplement: Supplementary file 7 — Supplementary Material [file JCMM-24-5274-s007.docx]

**Figure S1 PTBP1 expression is up-regulated in several common cancers.**

Analysis of PTBP1 expression and prognostic correlation in various of cancer tissues using TCGA datasets. (A-C, G-I) The expression level of PTBP1 in cancers and normal tissues in bladder cancer (BLCA), colon cancer (COAD), renal clear cell carcinoma (KIRC), hepatocellular carcinoma (LIHC), lung adenocarcinoma (LUAD) and gastric cancer (STAD). (D-F, J-L) In BLCA, COAD, KIRC, LIHC, LUAD and STAD, the relationship between PTBP1 expression level and prognosis. ****P*<0.001

**Figure S2 PTBP1 is up-regulated in osteosarcoma cell lines and cisplatin-resistant osteosarcoma cell lines.**

(A, B) The level of mRNA and protein of PTBP1 in osteosarcoma cell lines MG-63, U-2OS and human osteoblast cell line NHOst was measured by qRT-PCR and western blot. (C, D) The expression of PTBP1 in MG-63 and MG-63_CISR_ cells was measured by qRT-PCR and western blot. (E, F) The level of PTBP1 in U-2OS and U-2OS_CISR_ cells was measured by qRT-PCR and western blot. ***P*<0.01

**Figure S3 Transfection efficiency of sh-PTBP1 and si-SLC31A1 in CISR osteosarcoma cells.**

(A, B) qRT-PCR and western blot analysis confirmed the inhibition effect of sh-PTBP1 in MG-63_CISR_ and U-2OS_CISR_ cells. (C, D) qRT-PCR and western blot was used to determine the transfection efficiency of si-SLC31A1 in CISR osteosarcoma cells. **P*<0.05, ***P*<0.01, n.s is no significance

**Figure S4 The gene set enrichment analysis.**

(A, B) Gene Ontology analysis of differentially expressed genes in PTBP1 knockdown cells compared with control cells in MG-63_CISR_ (A) and U-2OS_CISR_ (B). (C, D) KEGG Pathway analysis of differentially expressed genes in MG-63_CISR_ (C) and U-2OS_CISR_ (D).
